# Impact of an Informational Video on Parental Choice of Tonsillectomy Technique

**DOI:** 10.1002/oto2.70306

**Published:** 2026-10-15

**Authors:** Amanda G. Baanante, Erin M. Gawel, Gaayathri Varavenkataraman, Annabella M. Nilon, Michele M. Carr

**Affiliations:** ^1^ Jacobs School of Medicine and Biomedical Sciences at the University at Buffalo Buffalo New York USA; ^2^ Department of Otolaryngology, Head and Neck Surgery Jacobs School of Medicine and Biomedical Sciences at the University at Buffalo Buffalo New York USA

**Keywords:** extracapsular tonsillectomy, intracapsular tonsillectomy, PS/QI

## Abstract

**Objective:**

To evaluate whether the use of an educational video explaining the differences between intracapsular and extracapsular tonsillectomy, as well as their respective risks and benefits, influences parental decision‐making in a pediatric otolaryngology clinic.

**Methods:**

Flyers with a quick response (QR) code linking to a video that explained intracapsular versus extracapsular tonsillectomy techniques and associated complications were posted in exam rooms of a pediatric otolaryngology clinic in July 2024. Parents were encouraged to view the video. Data were collected for tonsillectomies performed between May 2024 and January 2025, including clinic visit date, patient age and gender, parent at the visit, indication for tonsillectomy, and procedure chosen.

**Results:**

Of 214 patients, 133 (62%) had clinic visits before flyers were posted and 81 (38%) after. Before the flyers, only 24 (18%) chose intracapsular tonsillectomy, compared to 34 (42%) afterward (*P* < .001). Obstructive sleep apnea (OSA) or sleep disordered breathing (SDB) were the most common surgical indications (N = 200, 94%). Patient age and gender, parent at visit, and surgical indication were not significantly associated with the procedure chosen.

**Discussion:**

These findings suggest educational interventions can influence parental preference for tonsillectomy type. Providing accessible, comprehensible information may support more informed decision‐making, potentially improving surgical outcomes and parent satisfaction.

**Implications for Practice:**

Integrating concise educational tools into clinic visits offers a practical way to enhance communication and engage families in shared surgical decision‐making. These interventions may also standardize information delivery and reduce variability in how options are presented across providers.

Tonsillectomy is one of the most frequently performed surgical procedures in the pediatric population.^1^ The 2 most common indications in children are obstructive sleep apnea (OSA)/sleep disordered breathing (SDB) and recurrent tonsillitis.^2^ Extracapsular tonsillectomy, also referred to as complete or traditional tonsillectomy, involves the full removal of the tonsils along with their capsule and has historically been the standard approach.^3^ Intracapsular tonsillectomy, also known as partial tonsillectomy or tonsillotomy, has re‐emerged as an alternative technique. First described in the early 1900s, it gained broader acceptance nearly a century later, when Koltai detailed and refined the technique in the early 2000s.^4^ This approach involves partial removal of tonsillar tissue while preserving the capsule, reducing trauma to the surrounding musculature and vasculature.^5^

Posttonsillectomy hemorrhage (PTH) is a potentially serious postoperative complication, with a peak incidence between Days 7 and 10. Its severity can range from minor bleeding to life‐threatening events requiring urgent surgical intervention.^6^ A meta‐analysis by Lin et al reported that the rate of PTH after intracapsular tonsillectomy is low, at 1%.^7^ In a study comparing surgical techniques, Naidoo and Schlemmer found that intracapsular tonsillectomy was associated with a significantly lower rate of PTH (0.3%) when compared with extracapsular tonsillectomy (2.1%).^5^

Postoperative pain following tonsillectomy results from injury to the mucosa and associated sensory nerves.^8^ Russo et al demonstrated significantly lower postoperative pain scores in children undergoing intracapsular tonsillectomy when compared with those undergoing a extracapsular tonsillectomy.^9^ Similarly, in a study evaluating postoperative healthcare utilization after tonsillectomy, Kulich et al reported that patients in the intracapsular group had significantly fewer unplanned phone calls, 15‐day all‐cause ED returns, and 15‐day hospital readmissions. Specifically, among 9500 patients who underwent extracapsular tonsillectomy, 11.9% returned to the ED and 2.7% were readmitted, compared with 7.9% ED returns and 1.2% readmissions among 1705 patients who underwent intracapsular tonsillectomy. The majority of these postoperative contacts were related to oral bleeding and pain complaints.^10^

Long‐term outcomes of tonsillectomy have also been investigated. In a 10‐year follow‐up study of patients with SDB, Virkkuen et al found that there were higher rates of postoperative revisits and instances of revision surgery in the intracapsular tonsillectomy group. However, they found no differences between groups in patient satisfaction with the surgical outcome.^11^ Kisser et al also looked at the frequency of tonsillitis up to 5 years posttonsillectomy and found no significant difference in patients who had intracapsular and extracapsular tonsillectomy.^12^

A meta‐analysis by Gorman et al found that both intracapsular and extracapsular tonsillectomy led to improvement in OSA symptoms as measured by the OSA‐18 questionnaire.^13^ Likewise, Mabey et al demonstrated a significant improvement in the Clinical Assessment Score‐15, another office‐based questionnaire assessing pediatric SDB, for patients undergoing either intracapsular or extracapsular tonsillectomy, with no significant differences between groups.^14^

While intracapsular techniques, particularly with coblation, have shown promise in treating OSA with reduced surgical complications, adoption of the procedure has been limited. A major concern is the potential for tonsillar regrowth. Ahmarani et al utilized the Brodsky score to evaluate tonsillar regrowth following intracapsular tonsillectomy and found that about 2% of patients had tonsillar regrowth.^15^, ^16^

Informed decision‐making when selecting a surgical procedure is important. Individuals obtain, exchange, and learn about health information through various sources, including medical professionals, relatives, friends, advertising, media outlets, and online platforms including social media. Accurate multimedia education tools, such as informational videos, have emerged as a useful strategy to enhance patient and caregiver understanding.^17^

This quality improvement study was designed to evaluate whether the use of an educational video reviewing and comparing risks for intracapsular and extracapsular tonsillectomy, influences parental decision‐making in a pediatric otolaryngology clinic. Specifically, we assessed whether introducing a video‐based intervention, accessible via QR‐code flyers in examination rooms, influenced procedure selection. Given the increasing access to online health information, which may be misleading or inaccurate, our aim is to provide patients with a clear and accurate presentation of surgical options to support informed decision‐making.

## Methods

### Study Design and Setting

This quality improvement study was conducted in a pediatric otolaryngology clinic affiliated with a tertiary care children's hospital. The study was deemed quality improvement and was exempt from full board approval from the University at Buffalo Institutional Review Board.

All patients scheduled for tonsillectomy between May 2024 and January 2025 with one otolaryngologist were eligible for inclusion. Patients were identified retrospectively through the clinic electronic medical record system. Data collected included date of clinic visit, patient demographics, the indication for tonsillectomy (recurrent tonsillectomy or OSA/SDP), the procedure type selected by the parent or guardian (intracapsular or extracapsular tonsillectomy), and the identity of the parent or guardian attending the visit (mother, father, both, or other). No identifiable patient information was collected.

The study period was divided into 2 phases: preintervention (patients with clinic visits where a tonsillectomy was offered before the flyers were posted) and postintervention (patients with clinic visits after the flyers were posted). The intervention was introduced on July 9, 2024. All clinic dates prior to July 9 were considered preintervention.

### Intervention

The educational intervention consisted of printed flyers posted in all exam rooms. Each flyer contained a QR code linking to a short, custom‐made informational video designed for a lay audience.^18^ The video, presented in English with all spoken text transcribed, had a duration of 2 minutes and 36 seconds. The video outlined the key differences between extracapsular and intracapsular tonsillectomy, including indications, techniques, risks, and potential complications (Figure 1). The video was created by a medical student and a pediatric otolaryngologist. During clinic visits, the otolaryngologist encouraged parents/caregivers to scan the QR code and watch the video. This was in addition to the regular discussion between caregivers and the otolaryngologist regarding informed consent, options for tonsillectomy type, and risks and benefits of the procedure. All patients received counseling from the same otolaryngologist, which helped maintain consistency in how information was presented. The verbal discussion was not formally standardized or timed and was adapted based on caregiver questions and the natural flow of the clinical encounter. While the content of the discussion broadly aligned with the material presented in the educational video, the exact wording varied between visits.

**Figure 1:**
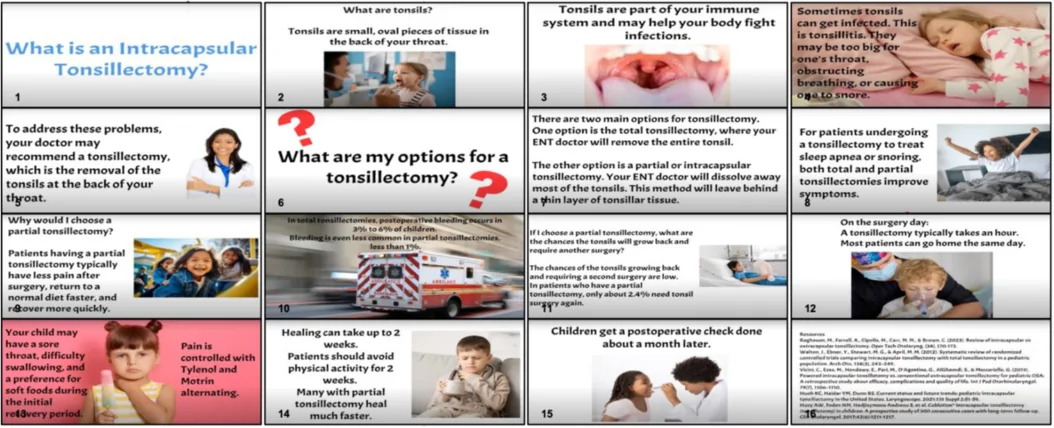
Video stills. Video stills from a QI educational module explaining tonsillectomy indications, intracapsular versus extracapsular techniques, associated risks, and potential complications for patient and caregiver education.

Parents could also access the video later via the same QR code for repeated viewing. Video viewing was optional, and no tracking of individual viewing was conducted. All patients were given the option between intracapsular and extracapsular tonsillectomy, except patients with asymmetric tonsils who were recommended extracapsular tonsillectomy.

### Outcomes

The primary outcome was the type of tonsillectomy chosen by the parent or guardian (intracapsular vs extracapsular). We assessed whether this choice was associated with patient demographics including age and gender, surgical indication, or the identity of the accompanying parent or guardian. Data were additionally stratified by intervention status (preintervention vs postintervention) to assess the impact of educational video on decision‐making.

### Statistical Analysis

All statistical analyses were conducted in SPSS (Version 30.0; IBM Corp, 2024). Continuous data were summarized with means and standard deviations (SD), and categorical data was summarized with frequency counts and percentages. Student *t* tests were used for comparisons of continuous data. Chi‐square tests were used to compare categorical data, unless 20% or more of the expected cell counts were less than 5, in which case Fisher exact tests were applied. Binomial logistic regression was used to estimate the likelihood of tonsillectomy choice based on predictor variables comprising patient age, patient gender, and flyer intervention. Statistical significance was defined as *P* < .05.

## Results

A total of 214 cases were identified, with a mean patient age of 6.1 years (SD = 3.0). In most cases, mothers attended the visit alone (N = 164, 76.6%). Obstructive sleep apnea (OSA) or sleep disordered breathing (SDB) were the most common surgical indications (N = 200, 93.5%). The most frequent tonsil size was +3 via the Brodsky scale (N = 105, 49.1%). 133 (62.1%) had clinic visits before flyers were posted and 81 (37.9%) after. Patient demographics and visit information are summarized in Table 1.

**Table 1:** Patient Demographics and Visit Data

Variable	Value
Child's age (years), mean (SD)	6.1 (3.0)
Gender of child, N (%)	
Male	113 (52.8)
Female	101 (47.2)
Parent at visit, N (%)	
Mother	164 (76.6)
Father	19 (8.9)
Both	27 (12.6)
Other	4 (1.9)
Sibling who had T&A, N (%)	
Yes	44 (20.6)
No	37 (17.3)
Unknown	133 (62.1)
Indication for surgery, N (%)	
OSA/SDB	200 (93.5)
Other	14 (6.5)
Tonsil size, N (%)	
+1	9 (4.2)
+2	40 (18.7)
+3	105 (49.1)
+4	58 (27.1)
Flyer intervention, N (%)	
Before flyer	133 (62.1)
After flyer	81 (37.9)
Tonsillectomy choice, N (%)	
Extracapsular tonsillectomy	156 (72.9)
Intracapsular tonsillectomy	58 (27.1)

Abbreviations: OSA/SDB, obstructive sleep apnea/sleep disordered breathing; SD, standard deviation; T&A, tonsillectomy and adenoidectomy.

Univariate analysis revealed a significant increase in the selection of intracapsular tonsillectomy following the flyer intervention, with 24 (18.0%) choosing this option before the intervention, compared to 34 (42.0%) after (*P* < .001). Patient age and gender, parent at visit, and surgical indication were not significantly associated with the choice of procedure (Table 2).

**Table 2:** Univariable Analysis of Factors Affecting Tonsillectomy Choice

Variable	Choice		*P* value
	Extracapsular tonsillectomy, N = 156	Intracapsular tonsillectomy, N = 58	
Child's age (years), mean (SD)	6.2 (2.5)	6.0 (2.7)	.726^a^
Gender of child, N (%)			
Male	80 (51.3)	33 (56.9)	.465^b^
Female	76 (48.7)	25 (43.1)	
Parent at visit, N (%)			
Mother	117 (75.0)	47 (81.0)	.588^c^
Father	14 (9.0)	5 (8.6)	
Both	21 (13.5)	6 (10.3)	
Other	4 (2.6)	0 (0.0)	
Sibling who had T&A, N (%)			
Yes	26 (16.7)	11 (19.0)	.744^b^
No	34 (21.8)	10 (17.2)	
Unknown	96 (61.5)	37 (63.8)	
Indication for surgery, N (%)			
OSA/SDB	143 (91.7)	57 (98.3)	.119^c^
Other^d^	13 (8.3)	1 (1.7)	
Tonsil size, N (%)			
+1	5 (3.2)	4 (6.9)	.356^b^
+2	29 (18.8)	11 (19.0)	
+3	81 (52.6)	24 (41.4)	
+4	39 (25.3)	19 (32.8)	
Flyer intervention, N (%)			
Before flyer	109 (69.9)	24 (41.4)	<.001^b^
After flyer	47 (30.1)	34 (58.6)	

Abbreviations: OSA/SDB, obstructive sleep apnea/sleep disordered breathing; SD, standard deviation; T&A, tonsillectomy and adenoidectomy.

^a^ Student *t* test.

^b^ Chi square.

^c^ Fisher exact.

^d^ Other indications included recurrent tonsillitis, tonsil stones, and tonsillar asymmetry.

Binomial logistic regression demonstrated a similar trend, with cases seen after the flyer intervention being 3.2 times (95% CI 1.7‐6.2, *P* < .001) more likely to choose intracapsular tonsillectomy compared to cases before the intervention, with no other variables significantly associated with increased odds of choosing this approach (Table 3).

**Table 3:** Binary Logistic Regression of Intracapsular Tonsillectomy Selection

Variable	Adjusted odds ratio	95% CI	*P* value
Age	1.022	0.905‐1.156	.722
Female [Ref. = Male]	0.972	0.513‐1.841	.930
OSA/SDB indication	5.258	0.626‐44.135	.126
After flyer [Ref. = Before flyer]	3.257	1.724‐6.154	<.001

Abbreviations: CI, confidence interval; OSA/SDB, obstructive sleep apnea/sleep disordered breathing; Ref. indicates reference category.

## Discussion

### Intracapsular Versus Extracapsular Tonsillectomy

The preference for intracapsular tonsillectomy among parents/caregivers as demonstrated in our study may reflect a desire to minimize postoperative pain, bleeding risk, and recovery time, even in light of the potential risk of tonsillar regrowth. This observed shift demonstrates the value of multimedia tools in supporting informed, evidence‐based surgical decision‐making.

### Multimedia Tools: Utility in Informed Consent and Patient Education

Multimedia tools offer an effective means of enhancing comprehension, especially among individuals with diverse learning styles and varying levels of health literacy. The integration of visual and auditory elements can make complex medical information more understandable and accessible for patients and caregivers. Several studies have demonstrated that multimedia education enhances understanding, outperforms written consent forms, supports comprehension across literacy levels, improves long‐term knowledge retention, and increases patient satisfaction throughout the informed consent process.^19^, ^20^, ^21^, ^22^ Additionally, multimedia educational tools can be accessed at any point during a patient's care, allowing patients and caregivers the opportunity to review information at home.

In pediatric surgery, where caregivers are the primary decision‐makers, these tools are especially valuable. Delemere and Maguire found that caregivers felt more confident and less frustrated when educational materials were provided by healthcare providers rather than sourced informally from friends or family, indicating greater trust in provider‐supplied content.^23^ Paton et al similarly found that parents reported significantly lower anxiety levels when multimedia tools were used to support informed consent for correction of pediatric hypertrophic pyloric stenosis.^24^ In another study, Shreyas et al demonstrated that multimedia videos were effective in reducing parental anxiety while improving comprehension and satisfaction.^25^

In otolaryngology, Newell et al found that adult patients often felt underprepared for surgery and the resulting lifestyle changes, highlighting the need for enhanced educational tools.^26^ Fang and Heckman reported that cancer patients using multimedia resources had greater increases in knowledge and fewer attitudinal barriers compared to those using traditional written materials.^27^ Likewise, Turkdogan et al found that multimedia education improved perioperative satisfaction in a randomized clinical trial involving various head and neck procedures.^17^

Taken together, these findings, and those of our study, emphasize the importance of integrating multimedia tools into clinical counseling to improve communication and decision‐making. Future studies should explore whether such tools impact postoperative outcomes, such as length of hospital stay, complication rates, and long‐term satisfaction.

### Parental and Patient Differences in Selection

Neither patient age nor gender was significantly associated with procedure selection, suggesting the educational video resonated with a broad cross‐section of caregivers. Although the sample size of male caregivers was too small for robust comparisons, previous studies suggest that mothers tend to report higher levels of preoperative concern. For example, Siddiqui et al, looked at parents whose children underwent tonsillectomy and found that 66% of mothers felt anxious about their child's procedure, compared to 34% of fathers.^28^ These findings are consistent with data from Selvi et al which found that mothers expressed higher anxiety levels than fathers during pediatric urologic surgery.^29^ This elevated concern may explain why parents/caregivers in our study were more likely to choose intracapsular tonsillectomy following the educational intervention, possibly due to its association with reduced pain and bleeding, even in the presence of a potential risk for regrowth.

### Limitations

This study has several limitations. This study was also conducted at a single academic‐affiliated clinic, which may limit generalizability. While some quality improvement studies can incorporate randomized designs, this project was not randomized, and we were unable to verify whether or to what extent parents engaged with the educational video or whether it directly influenced their decision making. We did not assess viewer comprehension or legibility of the video and therefore cannot determine whether the intervention improved understanding of tonsillectomy techniques or associated risks. No survey data were collected to evaluate caregiver perceptions of the video, and the educational content was not externally reviewed for potential bias in presentation. The verbal counseling with the otolaryngologist was not standardized or timed, and differences in caregiver questions or engagement following verbal versus video education were not evaluated.

Long‐term follow‐up was outside the scope of this project, and thus we cannot assess whether patients who chose intracapsular tonsillectomy remained satisfied with their decision or whether concerns such as tonsillar regrowth emerged. There are also broader barriers to implementing multimedia tools. Some patients or caregivers may lack access to the necessary technology or home internet, limiting their ability to engage with digital educational content. There is also a potential for language comprehension bias in the video, particularly if it is not in the viewer's native language or if it exceeds their reading comprehension level. This may reduce engagement or lead to misunderstandings about the information presented. Despite these limitations, our findings suggest that offering multimedia tools during clinic visits may influence parental decision‐making by providing an additional, accessible format for reviewing surgical indications, techniques, and associated risks. These resources may support more informed and confident decision‐making throughout the consent process.

## Implications for Practice

Overall, our study highlighted that a brief, targeted educational intervention can have a meaningful impact on parental decision‐making for their child's surgical procedure type. By giving patient caregivers the opportunity to view clear, accessible information, clinicians can promote more informed decision‐making and potentially enhance satisfaction and outcomes. Integrating concise educational tools into clinic visits also standardizes information delivery, reduces variability across providers, and encourages shared decision‐making between families and clinicians. Future research could evaluate long‐term satisfaction and the use of similar tools for other procedures or surgeries.

## Author Contributions

**Amanda G. Baanante,** substantial contributions to conception and design, acquisition of data, analysis and interpretation of data, drafting the manuscript and revising it critically for important intellectual content, provided final approval of the version to be published, agrees to be accountable for all aspects of the work in ensuring that questions related to the accuracy or integrity of any part of the work are appropriately investigated and resolved; **Erin M. Gawel,** Substantial contributions to acquisition of data, analysis and interpretation of data, drafting the manuscript and revising it critically for important intellectual content, provided final approval of the version to be published, agrees to be accountable for all aspects of the work in ensuring that questions related to the accuracy or integrity of any part of the work are appropriately investigated and resolved; **Gaayathri Varavenkataraman,** contributions to data acquisition, drafting the manuscript and revising it critically for important intellectual content, provided final approval of the version to be published, agrees to be accountable for all aspects of the work in ensuring that questions related to the accuracy or integrity of any part of the work are appropriately investigated and resolved; **Annabella M. Nilon,** contributions to revising the manuscript critically for important intellectual content, provided final approval of the version to be published, agrees to be accountable for all aspects of the work in ensuring that questions related to the accuracy or integrity of any part of the work are appropriately investigated and resolved; **Michele M. Carr,** contributions to conception and design, acquisition of data, analysis and interpretation of data, drafting the manuscript and revising it critically for important intellectual content, provided final approval of the version to be published, agrees to be accountable for all aspects of the work in ensuring that questions related to the accuracy or integrity of any part of the work are appropriately investigated and resolved.

## Disclosures

### Competing interests

None.

### Funding source

None.
